# Refinement strategy for antivenom preparation of high yield and quality

**DOI:** 10.1371/journal.pntd.0007431

**Published:** 2019-06-17

**Authors:** Tihana Kurtović, Maja Lang Balija, Marija Brgles, Dora Sviben, Monika Tunjić, Hrvoje Cajner, Martina Marchetti-Deschmann, Günter Allmaier, Beata Halassy

**Affiliations:** 1 University of Zagreb, Centre for Research and Knowledge Transfer in Biotechnology, Zagreb, Croatia; 2 University of Zagreb, Faculty of Mechanical Engineering and Naval Architecture, Ivana Lučića 5, Zagreb, Croatia; 3 Institute of Chemical Technologies and Analytics, TU Wien (Vienna University of Technology), Vienna, Austria; Universidad de Costa Rica, COSTA RICA

## Abstract

Antivenoms from hyperimmune animal plasma are the only specific pharmaceuticals against snakebites. The improvement of downstream processing strategies is of great interest, not only in terms of purity profile, but also from yield-to-cost perspective and rational use of plasma of animal origin. We report on development of an efficient refinement strategy for F(ab')_2_-based antivenom preparation. Process design was driven by the imperative to keep the active principle constantly in solution as a precautionary measure to preserve stability of its conformation (precipitation of active principle or its adsorption to chromatographic stationary phase has been completely avoided). IgG was extracted from hyperimmune horse plasma by 2% (*V*/*V*) caprylic acid, depleted from traces of precipitating agent and digested by pepsin. Balance between incomplete IgG fraction breakdown, F(ab')_2_ over-digestion and loss of the active principle's protective efficacy was achieved by adjusting pepsin to substrate ratio at the value of 4:300 (*w*/*w*), setting pH to 3.2 and incubation period to 1.5 h. Final polishing was accomplished by a combination of diafiltration and flow-through chromatography. Developed manufacturing strategy gave 100% pure and aggregate-free F(ab')_2_ preparation, as shown by size-exclusion HPLC and confirmed by MS/MS. The overall yield of 75% or higher compares favorably to others so far reported. This optimised procedure looks also promising for large-scale production of therapeutic antivenoms, since high yield of the active drug and fulfillment of the regulatory demand considering purity was achieved. The recovery of the active substance was precisely determined in each purification step enabling accurate estimation of the process cost-effectiveness.

## Author summary

Animal plasma-derived antivenoms constitute the most important therapy against snakebite envenoming. Nowadays this critical treatment has been faced by severe shortage due to low sustainability of current productions, which mostly affects developing countries as those suffering from highest morbidity and mortality rates. Antivenoms' safety and efficacy in clinical setting are highly dependent on manufacturing procedure. Its design should be guided by the tendency to refine immunoglobulin G from residual plasma proteins in only a few easy, simple and efficient purification steps, providing antibody-based product of acceptable physicochemical features and good recovery of protective activity. Here, we developed a compact, feasible and economically viable refinement strategy for antivenom preparation which looks promising for large scale production as well. Process design was driven by the imperative of keeping IgGs or F(ab')_2_ fragments constantly in solution in order to preserve stability of their conformations. In each of three main steps—caprylic acid precipitation for removal of contaminants, pepsin digestion of IgGs and chromatographic polishing of F(ab')_2_ active principle, optimal performance conditions were defined. As a result, preparation of completely homogenous and aggregate-free final product with over 75% yield was achieved in the most straightforward way. Also, the novel platform has been supported with process efficiency data, so accurate estimation of the cost-effectiveness is enabled.

## Introduction

Antivenoms prepared from hyperimmune animal plasma, mostly equine or ovine, are the only specific therapeutics for rapid counteracting post-snakebite pathophysiological manifestations. Although there are various well established downstream processing strategies that have been implemented into commercial scale production, optimisation of compact, high yielding and low-cost manufacturing procedures generating safe, efficacious and available immunotherapeutics is still of great interest.

Design of the ideal process should be guided by the tendency to refine immunoglobulin G from residual plasma proteins in only a few easy, simple and efficient purification steps, aiming for good recovery of neutralising activity and regulatory acceptable physicochemical characteristics of the product [1]. Quality of the final product depends also on immunisation scheme that should maximally boost humoral response, giving the highest possible titer of anti-venom antibodies. Many of so far developed strategies as the initial step employ salting-out procedure involving ammonium or sodium sulphate [2–6] that is associated with low purity profile of IgGs as well as excessive formation of aggregates [7, 8]. Both shortcomings can be prevailed by introducing caprylic acid as an alternative fractionation agent [7, 9, 10] which acts on the majority of plasma proteins without affecting IgG fraction by leaving it in solution and, consequently, preserving its conformational and structural stability [11, 12]. Refinement principles employing caprylic acid have been successfully implemented into preparations of a whole series of highly efficacious equine or ovine IgG-based antivenoms [13–17]. They have also been proven beneficial for purification of F(ab')_2_ derivatives [18, 19] and monoclonal antibodies [20].

Following IgG extraction some antivenom manufacturers perform enzyme-mediated separation of the Fc portion of IgG, because it is not important for the neutralisation activity, while its removal contributes to reduction of foreign protein quantity in the product intended for use in humans. It has been generally believed that the lack of the Fc fragment disables complement activation or inhibits the formation of immune complexes that are responsible for the onset of delayed hypersensitivity reactions [11, 21, 22]. However, poor physicochemical features of the product, *i*.*e*. turbidity, high content of IgG or contaminating protein aggregates, also exhibit detrimental impact, which was evidenced irrespective of the presence or absence of the Fc fragment [11, 12, 22]. Thus, its role in adverse reactions still remains unclear. Enzymatic cleavage can be performed either on unfractionated plasma [1, 18, 23] or isolated IgGs [1] as well as simultaneously with removal of unwanted proteins by caprylic acid precipitation [19]. Both F(ab')_2_ or Fab antivenoms have been successfully and widely used in snakebite management for decades [1], with the former ones being considered more clinically efficacious due to their slower elimination rate creditable for long lasting action [24]. Another advantage of F(ab’)_2_ fragments is associated with their divalent nature giving them ability to form multivalent immunocomplexes with venom antigens, just like whole IgG molecules. Such complexes may be removed by phagocytic cells, eliminating the toxins from relevant tissue locations. This mechanism does not operate in the case of Fab antibodies. The use of Fab fragments is often associated with recrudescence of envenomation signs, although their rapid distribution might represent desirable pharmacokinetic feature when dealing with venom toxins of comparable molecular weight.

Ion-exchange chromatography has been introduced into some refinement strategies as well, proving suitable for separation of F(ab')_2_ fragments from other plasma proteins under conditions preferring antibody adsorption on cation-exchange stationary phase material [18]. Additionally, it has been recognised also as a method of choice for the final polishing where an anion-exchange approach is favourably used [25]. Other chromatography techniques are also applicable, for example, purification of F(ab')_2_ fragments by means of affinity chromatography exclusively [26].

Our study aimed for integrating the most efficient segments of the existing technological knowledge from the field into a compact, feasible and economically viable purification strategy for preparation of equine plasma-derived antivenom based on F(ab')_2_ fragments. The main goal was to design an easily scalable sequence of purification steps in which desired IgG molecules or their fragments would be kept in solution throughout, as a precautionary measure against possible degradation/aggregation of the antibodies due to exposition to harsh condition (precipitation, binding to chromatographic support or high salt/low pH mediated elution) [27–30]. At the same time, effort was put into the preservation of highest process yield and fulfillment of the regulatory requirements concerning final product purity and aggregate content. The aim was also to precisely quantify the recovery of the active drug in each process step to enable accurate estimation of the cost-effectiveness of the designed procedure.

## Materials and methods

### Ethics statement

Adult mice (strain NIH Ola/Hsd, both sex, 18–20 g) for lethal toxicity neutralisation assay were purchased from the Institute of Immunology Inc. (Croatia). Animal use protocols were established in accordance to Croatian Law on Animal Welfare (2017) which strictly complies with EC Directive (2010/63/EU). Experiments involving animals were approved by the Croatian Ministry of Agriculture, Veterinary and Food Safety Directorate (UP/I-322-01/17-01/75, permission no. 525-10/0255-17-6). The approval is based on the positive oppinion of the National Ethical Commitee (EP 110/2017). During the test period mice were housed in a 12-h light/12-h dark cycle and at a constant temperature of 22 °C. A standard mouse diet (Mucedola srl., Italy) and water were supplied *ad libitum*. Animal monitoring for signs of pain, suffering and distress associated with procedure was performed following severity assessment protocol.

### Snake venom, plasma pools, animals and reagents

Crude venom of *V*. *a*. *ammodytes* (*Vaa*) and two pools of *Vaa*-specific hyperimmune horse plasma (HHP) were provided by the Institute of Immunology Inc. Caprylic acid, *o*-phenylenediamine dihydrochloride (OPD), iodoacetamide (IAA), dithiothreitol (DTT), bovine serum albumin (BSA), Tween 20, thimerosal, 2-(N-morpholino)ethanesulphonic acid (MES) monohydrate and Tris base were from Sigma-Aldrich, USA. Pepsin (from porcine gastric mucosa, 0.7 Ph. Eur. U/mg) was from Merck, Germany. Goat anti-horse F(ab')_2_ IgG conjugated with horseradish peroxidase (HRP) was from *antibodies-online*, Germany. All other chemicals used for preparation of buffers and solutions were from Kemika, Croatia.

### Purification of IgG standard for ELISA and model IgG substrate for pepsin digestion

Purification of IgG using protein A based affinity chromatography was performed by applying 12 mL of 2-fold diluted HHP per run on MabSelect Xtra columns (*V* = 2 × 1 mL, GE Healthcare, USA) with 20 mM Tris/HCl running buffer, pH 7.4, at a flow rate of 2 mL min^-1^. The bound antibodies were eluted with 20 mM citric acid, pH 2.3, diafiltrated into PBS, pH 7.0, and formulated with 0.3 M glycine. A highly purified IgG sample (eIgG) was used as standard in ELISA assay and as model substrate for preliminary optimisation of pepsin digestion.

### Optimisation of IgG purification by caprylic acid precipitation

HHP was incubated at 56 °C for 1 h. After centrifugation at 3,200 × *g* for 40 min and discarding the pellet, caprylic acid was added to 0.5 mL of supernatant in a dropwise manner so that final concentrations ranging from 1 to 9% (*V*/*V*) in 2-fold diluted reaction mixtures (*V* = 1 mL) were achieved. Precipitation was performed by vigorous stirring (750 rpm) at 23 °C for 1 h in thermomixer (Eppendorf, Germany), followed by sample centrifugation (2,800 × *g*, 45 min). IgG-enriched supernatant was collected and filtered through a cellulose acetate filter with a pore size of 5 μm (Sartorius, Germany). Minimal caprylic acid concentration giving the highest IgG purity and preserving yield, as preliminary determined, was chosen as optimal for the precipitation.

### Pepsin digestion optimisation

Preliminary optimisation of pepsin digestion was done using a model IgG substrate—highly pure IgG sample (eIgG) isolated from HHP by protein A based affinity chromatography. Generally, substrate aliquots (2 mg mL^-1^) were pH adjusted using 0.4 M HCl and tempered according to planned experiments. Pepsin solution (5 mg mL^-1^) in 0.15 M NaCl (saline) was added at different enzyme to IgG ratios (as specified in "Results" section) while gently mixing (350 rpm). The final volume of reaction mixture, prepared in saline, was 1 mL. Digestion was terminated at timed intervals with a 0.4 M NaOH solution until slightly acidic to neutral pH was achieved.

Initially, preliminary screening of four factors—pepsin to IgG ratio (1:300 or 10:300, *w*/*w*), duration of incubation (45, 60 or 90 min), pH of the reaction mixture (3.2 or 3.5), and temperature (20, 37 or 56 °C), was performed according to a general factorial experimental plan that consisted of a total number of 40 runs, four of which were assessed in duplicates. Since it was not possible to execute all runs simultaneously, their order was randomised to avoid systemic errors. We used a regression function model covering linear contribution of each factor, but also non-linear for selected experimental area.

In the second experiment duration of enzymatic reaction (X1) and pepsin to IgG ratio (X2), each at two levels (marked with minus (-) for the low and plus (+) for the high level), were further selected to study their impact on the digestion outcome. Their values were 1 or 3 h for X1 and 1:300 (*w*/*w*) or 10:300 (*w*/*w*) for X2. The full factorial design was employed resulting in 4 experimental runs, each performed in triplicate (2^2^ × 3). The main effect of each factor was calculated according to Eq (1),

$$E_{X}=\frac{2*\Sigma {\bar{Y}}^{+}{}_{j}}{n}-\frac{2*\Sigma {\bar{Y}}^{‑}{}_{j}}{n}$$ (1)

where index X represents factors 1 or 2, *n* is the total number of experimental runs (4) and ${\bar{Y}}_{j}$ are F(ab')_2_ yields obtained at - and + level of each factor. The significance of the given factors was determined by means of ANOVA using Statistica 13.4 software.

Fine-tuning of enzyme quantity with respect to IgG was tested by preparing reaction mixtures with a wide range of discrete pepsin concentrations (from 1:300 to 10:300, *w*/*w*) that were tested under conditions giving highest yield, according to results from previous experimental sets.

All subsequent experiments were performed using real process IgG substrate—IgG fraction from the optimised caprylic acid fractionation step, and examining one variable at a time. The common approach involved acidification to pH 3.2 and addition of pepsin solution in saline until desired enzyme to IgG ratio in a 1.5-fold diluted reaction mixture (*V* = 1 mL) was reached. Incubation was performed at 37 °C for 1.5 h. When optimal conditions were set, the procedure was scaled up 20-fold.

Samples from each experimental set were analysed by SDS-PAGE. The quantity of F(ab')_2_ fragments from reaction mixtures in which complete IgG cleavage occurred was measured by ELISA (detailed description is given in "ELISA assays for IgG and F(ab')_2_ content determination" section) and used for yield estimation [%]. For each run mean value and 95% confidence interval (CI) were calculated.

### Diafiltration steps

IgG-enriched supernatant following caprylic acid precipitation was diafiltrated into water or saline using Vivaspin device (Sartorius, Germany) with a 100 kDa molecular weight cut-off (MWCO) polyethersulfone membrane. F(ab')_2_ sample, as well as the commercial pepsin preparation employed for its preparation, were dialfiltrated into 20 mM MES buffer + 0.15 M NaCl, pH 5.0, on a membrane with a MWCO of 50 kDa. In each diafiltration step the buffer was exchanged by a factor of 8,000 ×.

### Development of flow-through chromatography for F(ab’)_2_ polishing

Chromatographic separation of pepsin from F(ab’)_2_ fragments was optimised on UNOsphere Q stationary phase (Bio-Rad, USA) in a batch mode with 20 mM MES with or without 0.15 M NaCl as binding buffer under varying pH conditions (from 4.0 to 6.0). Elution was performed with 1 M NaCl in the binding buffer. The starting material was crude F(ab')_2_—F(ab’)_2_ preparation obtained by pepsin digestion of caprylic acid fractionated IgGs (1 mL per 0.2 mg of stationary phase).

### F(ab’)_2_ polishing by flow-through chromatography

The sample (F(ab')_2_ obtained by pepsin digestion) was loaded (2 mL per run) to the pre-equilibrated CIM QA disk (*V* = 0.34 mL; BIA Separations, Slovenia) with 20 mM MES + 0.15 M NaCl binding buffer, pH 5.0, at a flow rate of 2 mL min^-1^ on ÄKTA chromatography system (GE Healthcare, USA). The absorbance was monitored at 280 nm. After collecting the flow-through fraction, the bound components were eluted from the column material with binding buffer containing 1 M NaCl.

### Pepsin activity

The enzymatic activity of pepsin was measured spectrophotometrically on Multiskan Spectrum instrument (Thermo Fischer Scientific, USA) using haemoglobin as substrate. Modified Ryle's protocol was followed [31]. Samples previously diafiltrated into 50 mM KCl, pH 2.0, using membrane with a MWCO of 10 kDa were prepared in 2-fold serial dilutions in duplicates. Aliquots of 40 μL were incubated with 200 μL of 2.5% (*w*/*V*) bovine haemoglobin solution previously acidified with 0.3 M HCl (60 μL). After 20 min at 37 °C the reactions were terminated by adding 1 mL of cold 4% (*w*/*V*) trichloroacetic acid. Non-degraded substrate was precipitated by centrifugation at 2,750 × *g* for 10 min and absorbance of the supernatants was measured at 280 nm. Blanks were obtained by omitting samples from reaction mixtures.

### SDS-PAGE and 2D gel electrophoresis

Purity of the IgG/F(ab')_2_ sample in each processing step was examined by SDS-PAGE analysis on 4–12% Bis-Tris gel with MES as running buffer under non-reducing conditions in an Xcell SureLock Mini-Cell, according to the manufacturer’s procedure (Invitrogen, USA). Staining was carried out with acidic Coomassie Brilliant Blue (CBB) R250 solution or, alternatively, with silver for detection of pepsin traces.

Isoelectric focusing, the first dimension of 2D gel electrophoresis, was performed in a ZOOM IPGRunner Mini-Cell (Invitrogen, USA) using immobilised pH gradient (IPG) strip (7 cm long, linear pH 3–10) (Invitrogen, USA) rehydrated with F(ab')_2_ sample (350 μg), according to the protocol provided by the manufacturer. The following step voltage protocol was applied: 200 V for 20 min, 450 V for 15 min, 750 V for 15 min and 2,000 V for 180 min. The second dimension utilised SDS-PAGE analysis of the protein focused on IPG strip that was reduced with 20 mM DTT and alkylated with 125 mM IAA prior its loading to 4–12% Bis-Tris gel. Obtained spots served as starting material for mass spectrometry (MS).

### MALDI-MS analysis

Excised protein spots obtained by 2D gel electrophoresis of F(ab')_2_ sample were prepared for MS analysis by in-gel trypsin digestion, as follows. Gel cuts were washed three times in water/acetonitrile (ACN) and once in NH_4_HCO_3_/ACN, dried under vacuum, reduced with 10 mM DTT (45 min at 56 °C) and then alkylated with 54 mM IAA (30 min at room temperature (RT) in the dark), both in 100 mM NH_4_HCO_3_. Following reduction and alkylation gel pieces were washed with 100 mM NH_4_HCO_3_ and ACN, dried and rehydrated in 1–10 μL of porcine trypsin solution (Roche, Germany) (10 ng of trypsin per estimated 1 μg of protein) for 45 min. Digestion was performed in 95% 50 mM NH_4_HCO_3_ and 5% ACN (*V*/*V*) overnight at 37 °C. Peptide extraction was repeated twice with 1% HCOOH/ACN 1:1 (*V*/*V*) and once more with water/ACN 1:1 (*V*/*V*). Pooled extracts were purified by C_18_ Zip-Tips (Millipore, USA), dried, dissolved again in 0.1% trifluoroacetic acid (TFA)/ACN 1:1 (*V*/*V*) and spotted on a stainless steel MALDI target (Bruker, Germany) after mixing with MALDI matrix (α-cyano-4-hydroxycinnamic acid (8 mg mL^-1^) in 0.1% TFA/ACN 1:1 (*V*/*V*)).

Measurements were performed on an ultrafleXtreme (Bruker, Germany) in positive, reflectron ion mode. The instrument is equipped with SmartBeam laser (355 nm), and the applied acceleration voltage was 8 kV in the positive ion mode. MS/MS spectra were obtained in the LIFT mode with the isolation of the monoisotopic peak. Obtained spectra were processed using FlexAnalysis (3.4.76.0) and BioTools (3.2. SR3). Identification searches were performed against NCBIprot database “Mammalia” (release 224, 02/2018 with 207,040,555 sequences) and against a contaminant database. Following parameters were used: precursor ion mass tolerance ± 200 ppm, product ion mass ± 1.0 Da, two missed trypsin cleavages and constant carbamidomethylation of Cys. Variable modifications such as N-acetylation, C-amidation, ammonia loss from N-terminal Cys, modification of N-terminal Gln to pyro-Glu, oxidation of Met, His or Trp and phosphorylation of Ser, Thr or Tyr were taken into account. Proteins were confidently identified by peptide mass fingerprint (PMF) and peptide sequencing if statistical scores were above respective threshold levels.

### Protein concentration determination

Throughout the isolation procedure total protein concentration was estimated spectrophotometrically by use of the Eq (2) [32],

$$\gamma \;\left[mg\;mL^{‐1}\right]=\left(A_{228.5\;nm}–A_{234.5\;nm}\right)\times f\times dilution\;factor$$ (2)

where Ehresmann's factor "*f*" for equine IgG of 0.2553 was used [33]. Appropriate dilution of each sample was independently prepared three times to obtain the mean value of the measured concentrations for further calculation of yield and purity.

### Size-exclusion chromatography

Size-exclusion chromatography (SEC), which was employed for monitoring of IgG/F(ab’)_2_ purity in all purification steps, as well as for analysis of pepsin preparation, was performed on TSK-Gel G3000SWXL column (7.8 × 300 mm) with 0.1 M phosphate-sulphate running buffer, pH 6.6, at a flow rate of 0.5 mL min^-1^ on Waters HPLC system (Waters, USA). The effluent was monitored at 280 nm. For determination of the IgG/F(ab')_2_ molecular weight, tyroglobulin (*M*_r_ 665,000), γ-globulin (*M*_r_ 150,000), ovalbumin (*M*_r_ 44,300) and ribonuclease A (*M*_r_ 13,700) were used as standards. Correction factor corresponding to deviation of molecular mass of analysed IgG, determined according to calibration curve, from its nominal molecular mass, was included in the calculation.

### ELISA assays for IgG and F(ab')_2_ content determination

ELISA for detection of specific antibodies in samples from HHP processing was performed by coating the microtiter plate with 100 μL / well of the venom coating solution (1 μL mL^-1^) in 50 mM carbonate buffer, pH 9.6, and left overnight at RT. After blocking with 0.5% (*w*/*V*) BSA in PBS with 0.05% (*V*/*V*) Tween 20 at 37 °C for 2 h, the starting plasma and samples from each purification step were added in 2-fold serial dilutions in duplicates and left overnight at RT. In IgG ELISA affinity purified IgG (eIgG) of precisely determined protein concentration served as a standard. In F(ab’)_2_ ELISA European viper venom antiserum (Zagreb antivenom, Institute of Immunology Inc.) was used as a standard. In the subsequent steps of ELISA, incubation with HRP-anti-horse F(ab׳)_2_ IgG (25,000-fold diluted) at 37 °C for 2 h occurred, followed by the addition of OPD (0.6 mg mL^-1^ solution) in citrate-phosphate buffer, pH 5.0. After 30 min of incubation in the dark, the enzymatic reaction was stopped with 1 M H_2_SO_4_ and the absorbance at 492 nm was measured.

Considering composition differences in subclass and/or venom-specific antibody distribution between each investigated sample and the standard, a recently developed principle for IgG or F(ab')_2_ estimation that uses sample-specific correction was applied [33]. Namely, for IgG determination a highly pure IgG-based product (pure IgG sample in Fig 9), which was processed from the respective HHP and precisely quantified, served as internal, sample-specific reference. Analogously, for F(ab’)_2_ quantification in process intermediates downstream of pepsin digestion step the purest F(ab’)_2_ preparation of precisely determined concentration (ultrapure F(ab’)_2_ in Fig 9) served as internal, sample-specific reference.

### Production process yields and sample purity calculation

Concentrations determined by ELISA assays were used for yield and purity calculations. IgG yield was calculated as: [(*γ*(IgG) × dilution factor) / *γ*(IgG) in starting material] × 100%. Purity of each IgG intermediate was expressed as: (*γ*(IgG) / *γ*(protein)) × 100%. F(ab')_2_ yield was calculated as: [(*γ*(F(ab')_2_) × dilution factor) / (*γ*(IgG) in starting material × 0.67)] × 100%. Purity of each F(ab')_2_ intermediate and final product was expressed as: (*γ*(F(ab')_2_) / *γ*(protein)) × 100%. Additionally, SEC monitoring for purity profiling throughout the manufacturing procedure was included also.

### ED_50_ test

The potential of HHP and pure IgG/F(ab')_2_ preparation to neutralise the venom’s lethal toxicity was determined by the lethal toxicity neutralisation assay in mice, as previously described [34]. The lethal toxicity neutralisation potency (*R*) was expressed as the number of LD_50_ venom doses that can be neutralised by 1 mL of undiluted sample and calculated by the Eq (3),

$$R=\left(Tv‐1\right)/ED_{50}$$ (3)

where Tv represents the number of LD_50_ venom doses inoculated per mouse. *R*-value was used as a measure of the protective efficacy of each sample. Specific activity (LD_50_ mg^-1^) was expressed as ratio of *R*-value and either active principle (IgG or F(ab')_2_) or total protein concentration.

### Data analysis

Unless stated otherwise, the results of each analysis are expressed as the average of *n* measurements, with uncertainty of measurements expressed as 95% CI. Number of measurements for each analysis (*n*) is given.

## Results

### Optimisation of IgG purification by caprylic acid precipitation

**Fig 1:**
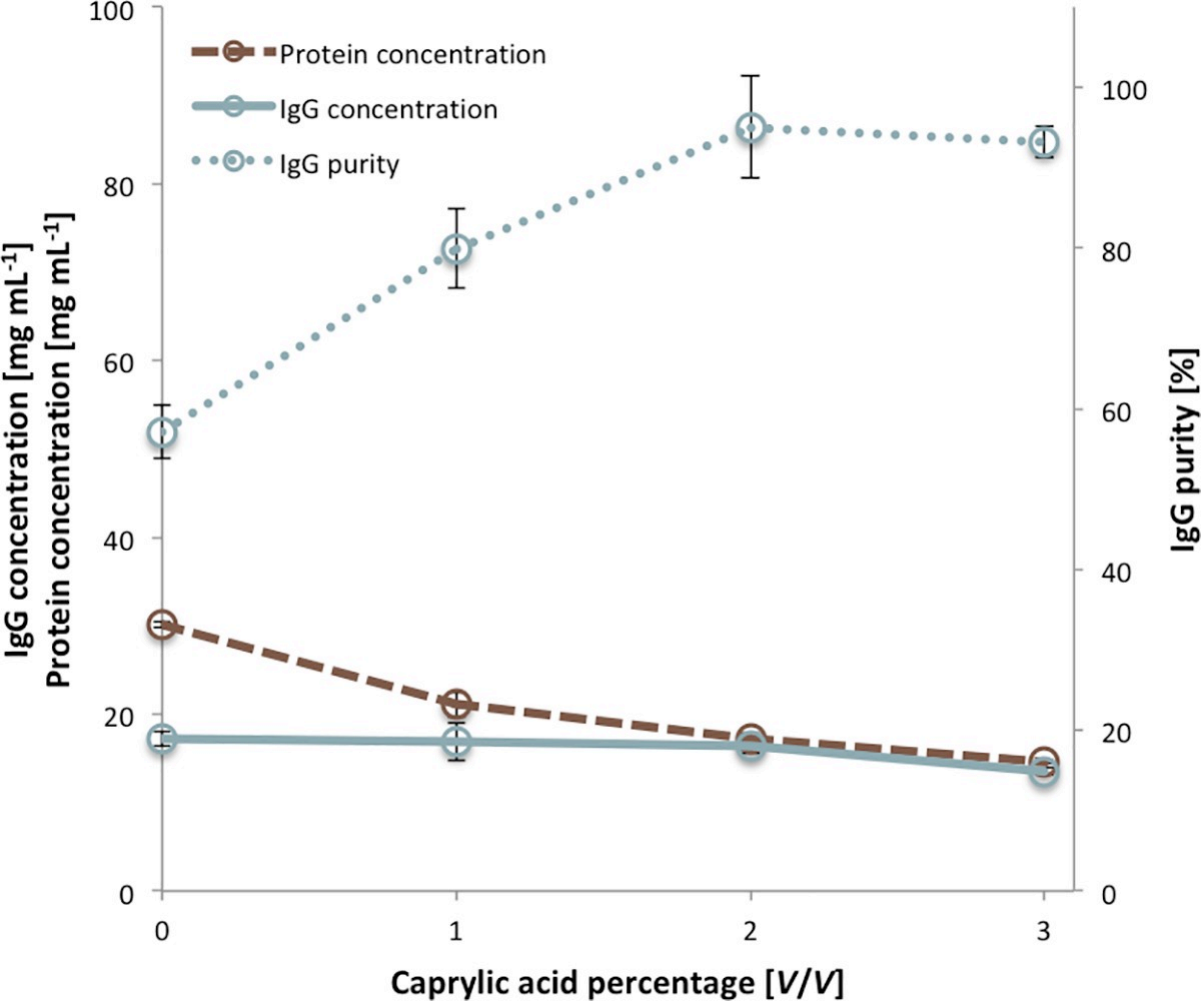
Preliminary determination of optimal caprylic acid concentration for precipitation step of the purification protocol. Results (total protein concentration, IgG concentration and purity) are given as mean +/- 95% CI (*n* = 3).

Initially, heat-treated (defibrinogenated) HHP was fractionated by caprylic acid (1–9%, *V*/*V*). As became evident after centrifugation, concentrations higher than 3% negatively affected visual appearance of the supernatant, causing excessive turbidity and formation of a non-precipitable layer on top of the aqueous phase. Thus, only samples fractionated by lower concentrations were further analysed for IgG and total protein content. According to ELISA-based calculations, the investigated range of caprylic acid concentrations (1–3%) did not precipitate IgG molecules, leaving them completely in supernatant, while the lowest one significantly impaired their purity (Fig 1). At 2% caprylic acid, precipitation of almost all unwanted proteins occurred. Higher concentration did not exhibit any obvious beneficial effect.

**Fig 2:**
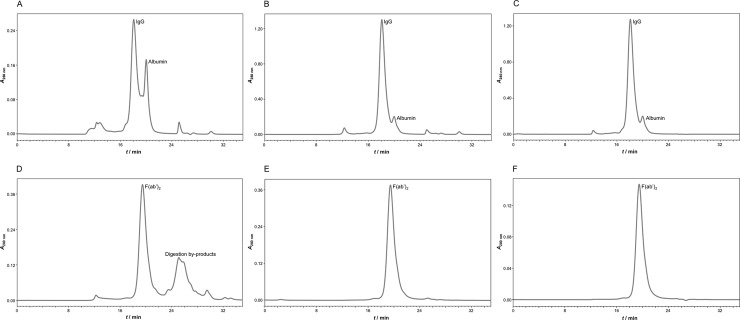
The assessment of purification steps by size-exclusion chromatography. Analysis was performed on TSK-Gel G3000SWXL column (7.8 × 300 mm) with 0.1 M phosphate-sulphate running buffer, pH 6.6, at a flow rate of 0.5 mL min^-1^. Heat-treated plasma (A). IgG fraction—supernatant from 2% caprylic acid precipitation before (crude IgG; B) and after diafiltration using a 100 kDa membrane (pure IgG; C). F(ab')_2_ fraction produced by pepsin digestion of IgG preparation before (crude F(ab')_2_; D) and after diafiltration using a 50 kDa membrane (pure F(ab')_2_; E). Ultrapure F(ab')_2_ preparation—flow-through fraction from anion-exchange chromatography performed at pH 5.0 (F). Detection: UV at 280 nm.

**Fig 3:**
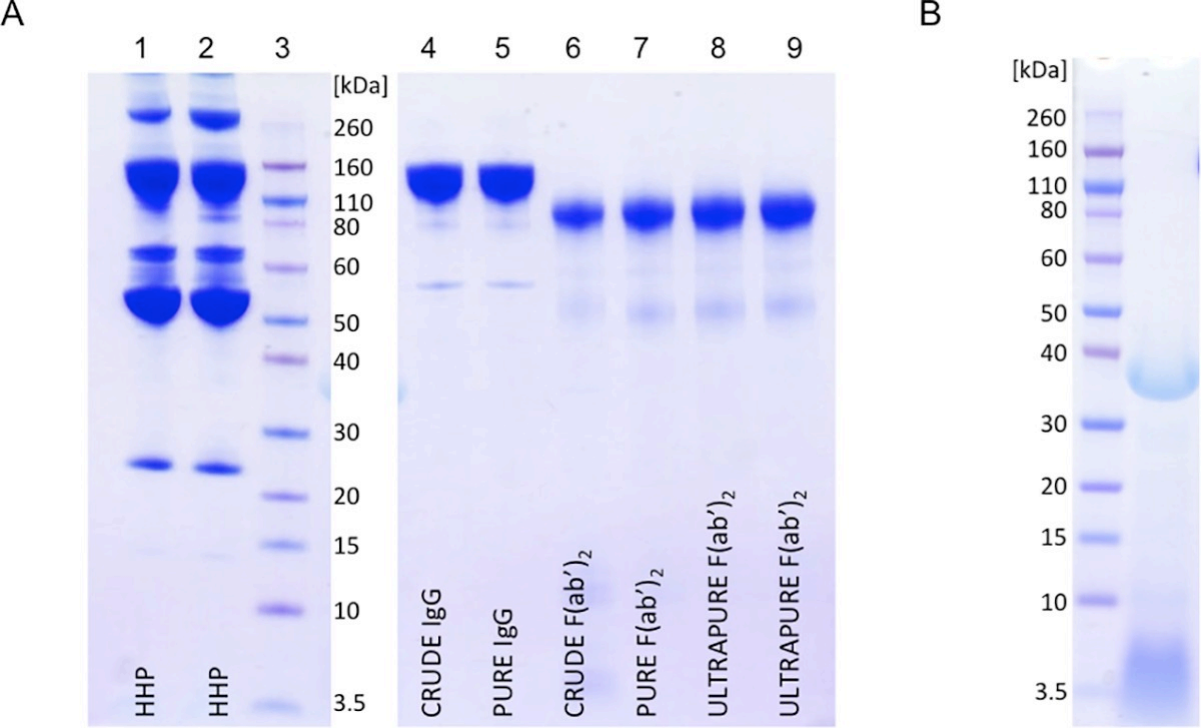
**SDS-PAGE analysis of representative samples from purification process (A) and pepsin preparation (B).** Lane 1 and 2, hyperimmune plasma pools; lane 3, molecular weight standards; lane 4, IgG fraction obtained by caprylic acid precipitation (crude IgG); lane 5, IgG fraction after diafiltration (pure IgG); lane 6, F(ab')_2_ fraction produced by pepsin digestion of IgG preparation (crude F(ab')_2_); lane 7, F(ab')_2_ preparation after diafiltration (pure F(ab')_2_); lanes 8 and 9, F(ab')_2_ preparation polished using CIM QA chromatography (ultrapure F(ab')_2_). The analysis was done on 4–12% gel under non-reducing conditions. Staining was performed with CBB R250.

Caprylic acid at a volume ratio of 2% was identified to be the minimal quantity required for preservation of yield and acquisition of high purity so it was chosen as optimal for HHP fractionation. Based on ELISA results, 2% caprylic acid enabled the preparation of crude IgG sample almost without any losses and at > 80% purity (Table 1). SEC analysis of samples from two initial steps, heat-treatment and precipitation, confirmed significant reduction of the non-IgG protein content in supernatant as well (Fig 2A and 2B). SDS-PAGE profiles are shown in Fig 3A (lanes 1 or 2 and 4). The molecular mass of equine IgG, assessed by SEC, was 153.5 ± 1.5 kDa (*n* = 78).

**Table 1:** Purities and yields of the intermediates and the final product obtained by developed downstream processing protocol.

PROCESSING STEP	PRODUCT	IgG / F(ab')_2_ PURITY [%]	IgG / F(ab')_2_ YIELD [%]*	OVERALL IgG / F(ab')_2_ YIELD [%]**
	Plasma	47.8 ± 1.6 (*n* = 41)	n.a.	n.a.
Heat denaturation	Thermally treated plasma	46.9 ± 3.0 (*n* = 9)	91.6 ± 2.4 (*n* = 8)	91.6 ± 2.4 (*n* = 8)
Caprylic acid precipitation	Crude IgG	81.3 ± 2.2 (*n* = 6)	97.3 ± 0.7 (*n* = 6)	89.6 ± 3.6 (*n* = 8)
Diafiltration	Pure IgG	89.0 ± 1.4 (*n* = 5)	102.3 ± 3.75 (*n* = 8)	91.5 ± 2.0 (*n* = 7)
Pepsin digestion	Crude F(ab')_2_	56.3 ± 6.4 (*n* = 7)	92.0 ± 11.3 (*n* = 7)	84.1 ± 10.5 (*n* = 7)
Diafiltration	Pure F(ab')_2_	100.3 ± 5.2 (*n* = 7)	92.6 ± 6.9 (*n* = 8)	78.6 ± 3.0 (*n* = 6)
Anion-exchange HPLC	Ultrapure F(ab')_2_	100.0 ± 2.8 (*n* = 6)	100.0 ± 2.5 (*n* = 23)	77.0 ± 2.3 (*n* = 6)

Purity and yield were calculated according to ELISA. Results are given as mean +/- 95% CI.

* Yield of each processing step obtained during optimisation of refinement strategy.

** Overall yield obtained by developed manufacturing protocol that was independently performed several times on two plasma pools by two analysts.

### Pepsin characterisation

**Fig 4:**
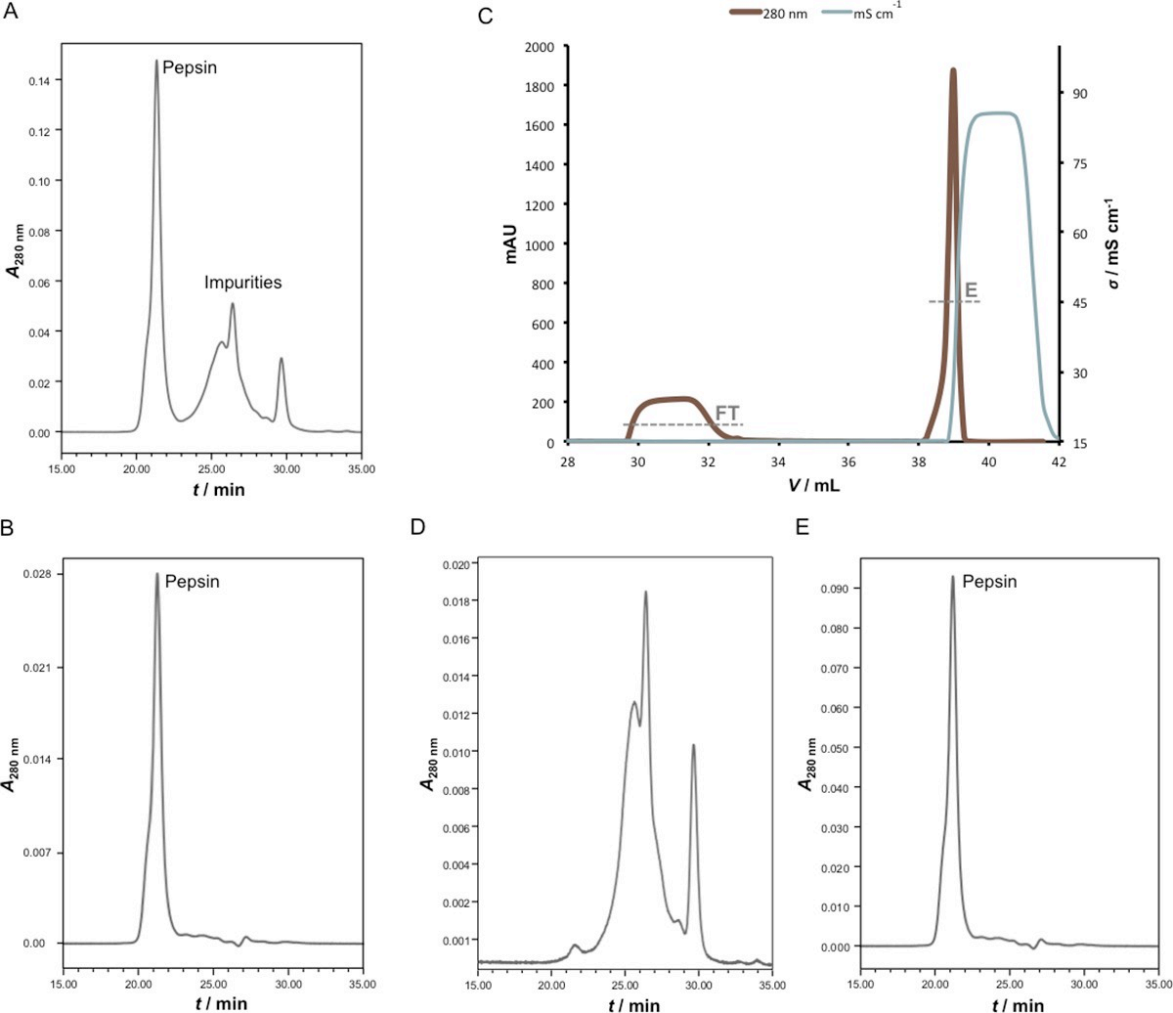
Verification of pepsin removal by the final polishing procedure. SEC analysis of pepsin sample on TSK-Gel G3000SWXL column (7.8 × 300 mm) with 0.1 M phosphate-sulphate running buffer, pH 6.6, at a flow rate of 0.5 mL min^-1^ prior (A) and post-diafiltration on a 50 kDa membrane (B). Anion-exchange chromatography of pepsin sample on CIM QA disk *(V* = 0.34 mL) with MES + 0.15 M NaCl buffer, pH 5.0, at a flow rate of 2 mL min^-1^ (C). Enzymatically active material was retained on the column and it was subsequently eluted with 100% yield. Manufacturing by-products that affect pepsin purity lacked capability of binding. Size-exclusion chromatography of flow-through (D) and elution fraction (E) from anion-exchange chromatography in (C). Detection: UV at 280 nm.

Commercial pepsin preparation involved in the manufacturing procedure had 7 times lower total protein concentration in comparison to the one derived from the weighted mass. In addition, when analysed by SDS-PAGE, notable contamination of the enzyme with autodigestion and/or manufacturing by-products was observed (Fig 3B). SEC profile corroborated the obtained results concerning composition of the enzyme preparation, revealing that only 48.4 ± 0.8% (*n* = 6) of the whole protein content actually corresponds to pepsin with a molecular weight of 35.9 ± 0.1 kDa (*n* = 6) (Fig 4A). The majority of contaminating proteins/peptides was approximately 10 kDa or less. As demonstrated, their complete removal could be achieved through diafiltration on a 50 kDa membrane (Fig 4B) which at the same time leads to the loss of 40% of pepsin content.

### Optimisation of pepsin digestion

#### Preliminary screening of digestion conditions

**Fig 5:**
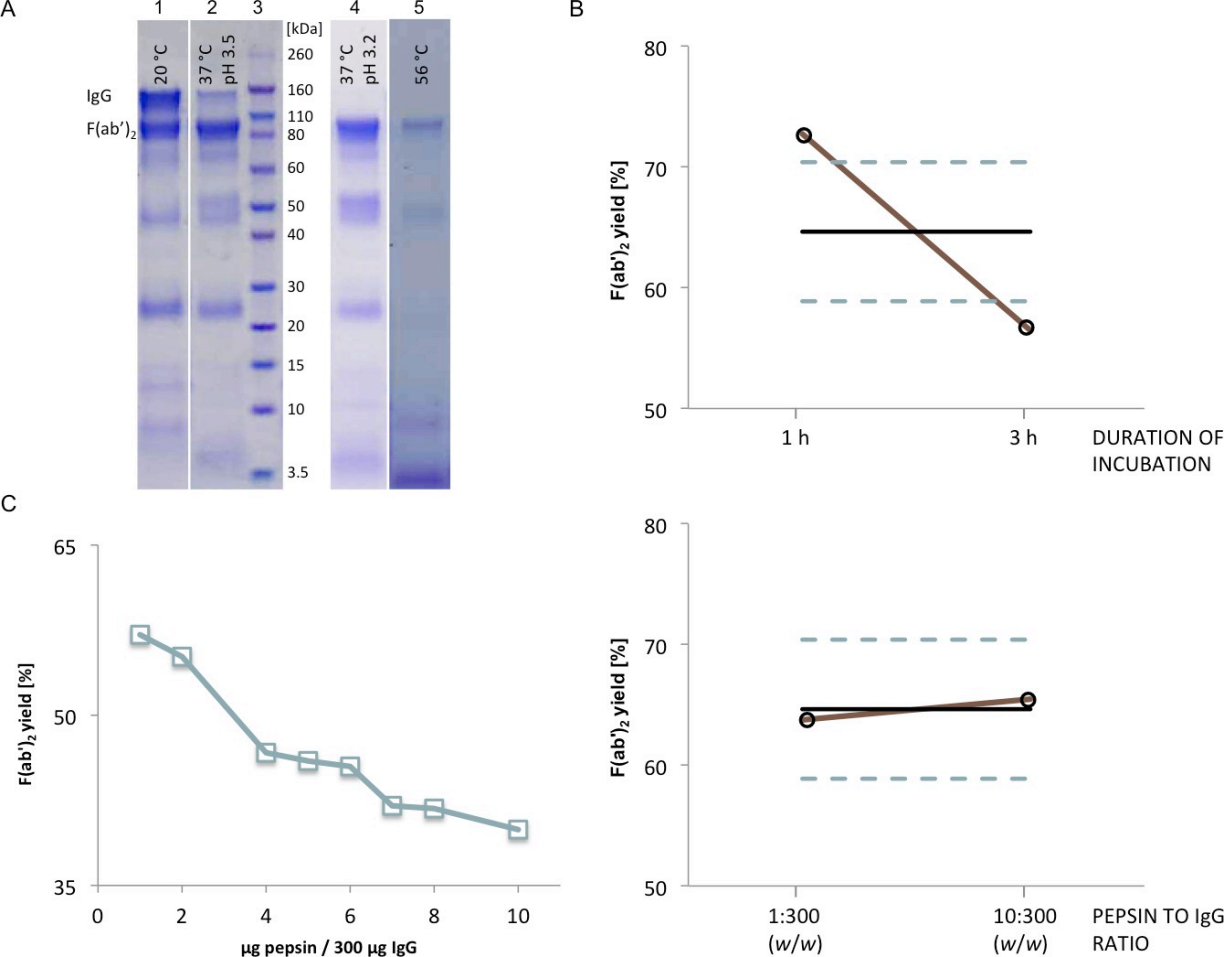
Preliminary screening of pepsin digestion conditions. (A) SDS-PAGE analysis of representative samples obtained by pepsin digestion of highly pure IgG sample (eIgG) on 4–12% gel under non-reducing conditions. Lane 1, typical digestion pattern after incubation at 20 °C; lane 2, typical digestion pattern after incubation at 37 °C when pH was set to 3.5; lane 3, molecular weight standards; lane 4, typical digestion pattern after incubation at 37 °C when pH was set to 3.2; lane 5, typical digestion pattern after incubation at 56 °C. In all lanes samples obtained by pepsin to IgG ratio of 10:300 (*w*/*w*) during 1.5 h-long incubation were presented. Staining was performed with CBB R250. (B) Optimisation of pepsin digestion with respect to duration and pepsin to IgG ratio studied according to full factorial experimental design. Mean yield (ο) of active drug obtained at higher and lower level of each experimental factor (X1—duration of enzymatic reaction; X2—pepsin to IgG ratio) in comparison to mean value (full line) and 95% CI (dashed lines) from the overall set of experiments. (C) Impact of pepsin to IgG ratio on F(ab')_2_ yield measured by ELISA assay.

Pepsin digestion was preliminary optimised on model IgG sample prepared by affinity chromatography (eIgG). Its purity was 98%, as determined by SEC. In the first experiment, pepsin to IgG ratio (1:300 or 10:300, *w*/*w*), duration of incubation (45, 60 or 90 min), pH of the reaction mixture (3.2 or 3.5) and temperature (20, 37 or 56 °C) were tested in 40 experimental runs according to the fractional factorial design of experiments. Quantitative data analysis with appropriate statistics, as originally planned, was not possible, since in some experimental runs the digestion was not completed, and we were not able to separately quantify IgGs and nascent F(ab’)_2_ fragments present in the same sample. However, the experimental runs were analysed by SDS-PAGE and conclusions were drawn from differences in their protein pattern and intensity of detected bands. Incubation at 20 °C (RT) (12 runs), which was included in the analysis due to possible co-performance of caprylic acid precipitation together with enzymatic cleavage, did not support digestion irrespective of the reaction mixture's pH or pepsin concentration (Fig 5A, lane 1). Similarly, at 37 °C, the standard temperature for most enzymatic reactions, a great fraction of eIgG sample remained intact when pH 3.5 was employed (8 runs) (Fig 5A, lane 2). On the other hand, by adjusting pH to 3.2, hydrolysis at 37°C was fully finalised after either 45 (at enzyme to IgG ratio of 10:300, *w*/*w*) or 90 min (at enzyme to IgG ratio of 1:300, *w*/*w*) (Fig 5A, lane 4). Incubation at 56 °C (12 runs), which was chosen with the idea of eventual simultaneous performance of HHP defibrinogenation and pepsin digestion, proved inappropriate (Fig 5A, lane 5). It provoked further F(ab')_2_ degradation—very faint bands at 100 kDa or their complete absence were observed. Yield was lower than 27%. All experimental runs with fully completed IgG hydrolysis (performed at pH 3.2 and 37 °C) (8 runs) were subjected to ELISA-based quantification of F(ab')_2_ fragments, giving yield between 65 and 75%.

In the second experiment, set at pH 3.2 and 37 °C, only duration of incubation (1 or 3 h) and pepsin to IgG ratio (1:300 or 10:300, *w*/*w*) were investigated according to the full factorial design. The whole IgG population underwent cleavage in all experimental runs, which was a necessary prerequisite for ELISA-based quantification of F(ab')_2_ fragments. Their yields varied between 55 and 73%. The recovery was negatively influenced by the reaction time (longer incubation lowered yields) (Fig 5B). The main effect estimate *E*_X_ for factor X1 (duration of incubation) was -16.30 and 1.73 for factor X2 (pepsin to IgG ratio). The statistical analysis showed that only X1 had significant impact on the response variable (*p* < 0.0001). The possibility that its effect was a result of coincidence is lower than 0.0034%. Factor X2 and interaction X1*X2 had no significant impact on response.

First two experiments of digestion conditions on a model substrate, eIgG, revealed that its full cleavage with the highest yield was achieved when performance conditions were: pH 3.2, 37 °C and a reaction time of 60 to 90 min. For the fine-tuning of the enzyme concentration a series of pepsin to IgG ratios from 1:300 to 10:300 (*w*/*w*), ten in total, was investigated. The experiment indicated gradual decline of F(ab')_2_ concentration with the increase of enzyme content (Fig 5C). Thus, pepsin to IgG ratio of 1:300 (*w*/*w*) proved the best among tested when performing enzymatic cleavage on highly pure substrate.

#### Pepsin digestion on IgG obtained by caprylic acid precipitation

The efficiency of established conditions for digestion step was verified on the IgG fraction obtained by 2% caprylic acid precipitation (crude IgG). Due to its lower purity (80%) in comparison to eIgG prepared by affinity chromatography (98%), initially a slightly higher enzyme to protein ratio was prepared, 2:300 (*w*/*w*) respectively.

**Fig 6:**
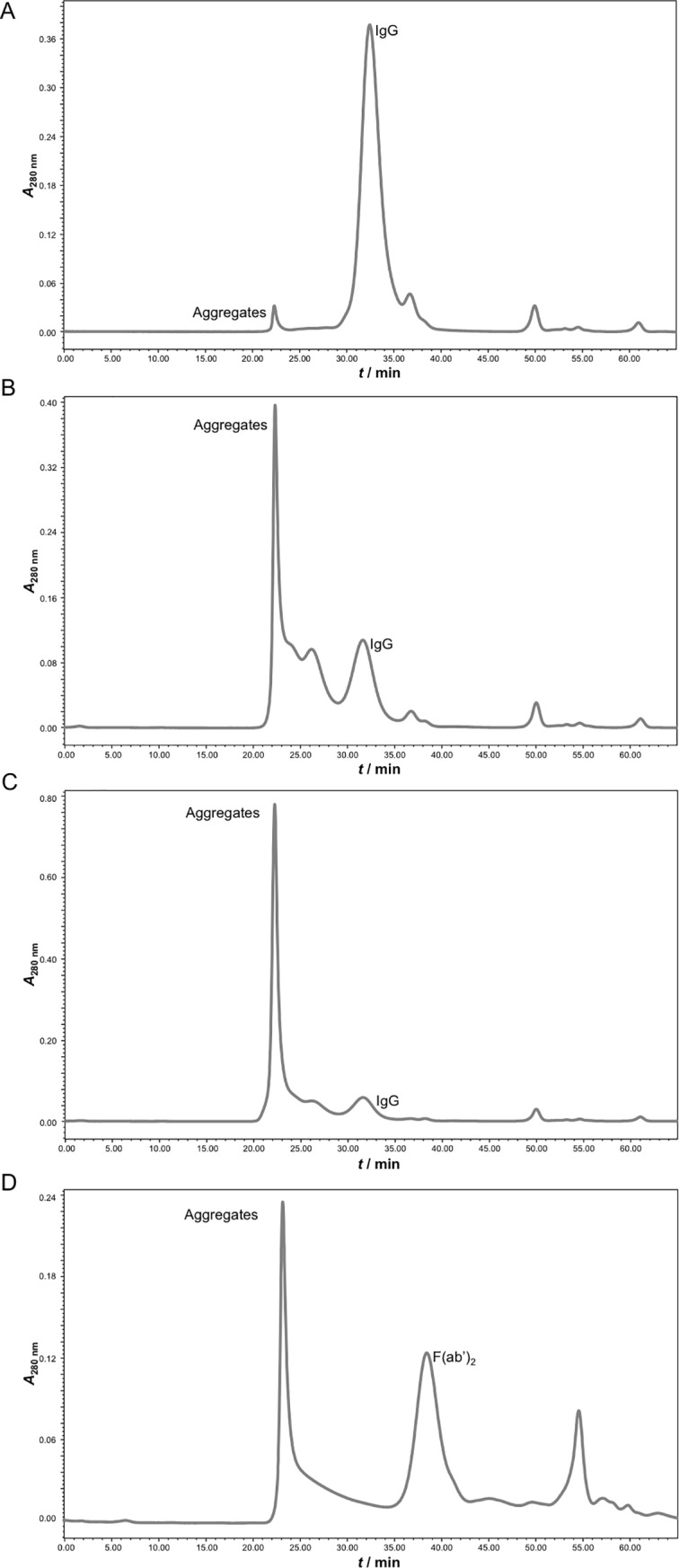
The assessment of IgG/F(ab')_2_ aggregation by size-exclusion chromatography. Analysis was performed on TSK-Gel G3000SWXL column (7.8 × 300 mm) with 0.1 M phosphate-sulphate running buffer, pH 6.6, at a flow rate of 1 mL min^-1^. IgG substrate—supernatant from 2% caprylic acid precipitation before diafiltration (A). IgG fraction acidified to pH 3.2 in the presence of 2% caprylic acid (*V*/*V*) and incubated at +4 °C (B) or 37 °C (C). F(ab')_2_ fraction produced by pepsin digestion in the presence of caprylic acid (D). Detection: UV at 280 nm.

Firstly, the mock experiment was performed in which the behavior of IgGs was studied in the absence of pepsin, but under conditions used in digestion step (pH 3.2, 37 °C). The starting material, crude IgG from caprylic acid precipitation, was composed of monomers mostly, as revealed by SEC, and a peak corresponding to aggregates comprised only 3.0 ± 1.1% (*n* = 6) of the total area under the chromatogram (Fig 6A). When exposed to acidic environment a rather high IgG portion became prone to aggregation. Namely, if the incubation was performed at 4 °C, SEC revealed around 70% of aggregates near the exclusion limit (> 500,000 Da) (Fig 6B). At 37 °C as default temperature for pepsin digestion more than 80% of the total protein content was aggregated (Fig 6C). Raising the pH to 4.0, 5.5 and 7.0 with 0.4 M NaOH, 1 M Tris base or 0.5 M Na_2_HPO_4_, common approach for enzyme inactivation, did not impact the extent of the phenomenon. These findings demonstrate that the type of alkalising reagent has no bearing on the results. According to SEC, the greater difference between initial and adjusted pH was associated with the more prominent multimers of even higher molecular weight. As potential IgG stabilisers glycine (0.3 or 0.5 M), sucrose (7.5 or 10%, *w*/*w*) and urea (0.5 or 1 M) were tested, but without success. Moreover, the aggregation was irreversible since it could not be alleviated by subsequent elimination of caprylic acid by diafiltration. Similarly, a crude IgG sample incubated with pepsin under standardised conditions (enzyme to IgG ratio of 2:300 (*w*/*w*), pH 3.2, 37 °C, 90 min) gave rise to F(ab')_2_ fragments with almost half of them aggregated (Fig 6D).

Furthermore, a crude IgG sample (supernatant after precipitation step) was depleted from remaining caprylic acid by diafiltration on a 100 kDa membrane either into water or 0.15 M NaCl. ELISA-determined purity was enhanced to almost 90% through elimination of small molecular weight proteins/peptides, as evident from SEC profile (Fig 2C). Diafiltrated preparation was assigned as pure IgG sample. Its aggregate content was 1.6 ± 0.3% (*n* = 6) and somewhat lower in comparison to that of the crude IgG sample. Despite caprylic acid removal, when a pure IgG sample was exposed to acidic environment, aggregation phenomenon reappeared, but to a lesser extent—multimers varied from 13 to 43%, depending on the pH chosen for terminating the "enzymatic reaction". Following pepsin digestion in water, an aggregate-free preparation was obtained, but substrate hydrolysis was incomplete. When 0.15 M NaCl was employed, the whole IgG population underwent cleavage, resulting in F(ab')_2_ fragments as dominant product (approximately 56%) of expected molecular weight (97.5 ± 1.5 kDa, *n* = 77) (Fig 2D). Pepsin digestion resulted in crude F(ab')_2_ sample. Since saline proved to be more supportive for hydrolysis than water, the same formulation was used for all previous steps requesting dilution or washing out procedures. At an enzyme to IgG ratio of 2:300 (*w*/*w*) in a crude F(ab')_2_ sample aggregates comprised about 5%. By adjusting to 4:300 (*w*/*w*) their content was lowered to 0.3%.

Under optimised conditions no significant loss with respect to IgG sample diafiltrated into 0.15 M NaCl (pure IgG) as input material for digestion step occurred (Table 1). The average yield in regards to IgG in starting plasma was 84%. The expected mass reduction of 67% due to the Fc part removal was taken into account. The calculated ratio of 0.64 for SEC-determined molecular weights of F(ab')_2_ and IgG only slightly deviated from the nominal value used for monitoring of process efficiency (0.67).

### Optimisation of conditions for pepsin removal

**Fig 7:**
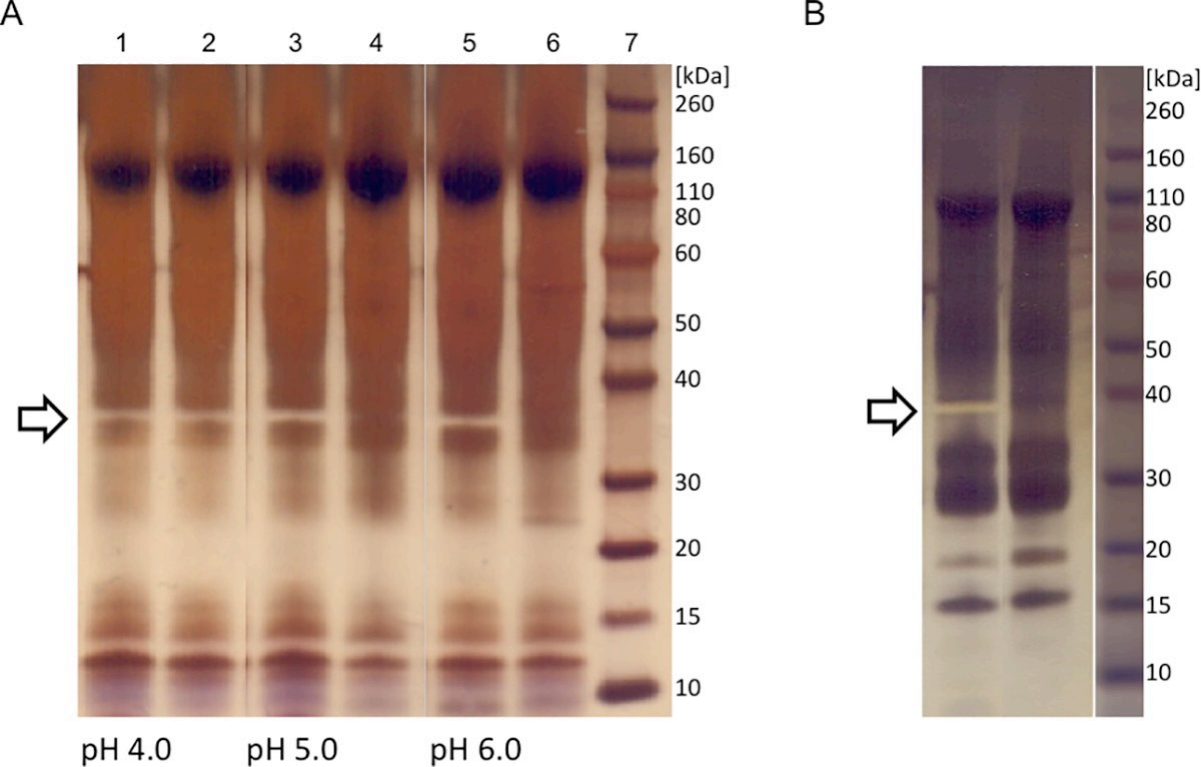
SDS-PAGE analysis of F(ab')_2_ preparation after flow-through chromatography under different pH conditions and detection of pepsin traces. (A) Detection of pepsin in Unosphere Q fractions by "negative" silver staining method. Lanes 1, 3 and 5, crude F(ab’)_2_ as starting material; lanes 2, 4 and 6, unbound fractions at pH 4.0, 5.0 and 6.0, respectively. (B) SDS-PAGE analysis of F(ab’)_2_ preparation before (left lane) and after CIM QA chromatography (right lane) at pH 5.0. Molecular mass markers are at right side. Staining was performed with AgNO_3_. "Negatively" silver stained bands corresponding to pepsin are marked.

Study in a batch mode on UNOsphere Q stationary phase for optimisation of the chromatographic separation of pepsin from F(ab’)_2_ fragments revealed pH 5.0 as the most convenient for achieving a good balance between its removal by adsorption to anion-exchange support and retention of the active principle in solution with minimal loss. So it was further used for the final polishing step. Namely, each of the investigated pH values (4.0, 5.0 or 6.0) resulted in good F(ab')_2_ fragment yield of minimum 85% (Table 2). The presence of 0.15 M NaCl in the binding buffer had a slightly positive impact on their recovery in the unbound fraction, at the same time unaffecting quantity of the pepsin retained on the support. Although the highest F(ab')_2_ yield was achieved under conditions employing pH 4.0, contaminating traces of pepsin were confirmed in the unbound fraction, as concluded from the presence of a ''negatively'' silver stained band at position corresponding to its molecular weight (Fig 7A) [35].

**Table 2:** Characterisation of the unbound fractions following incubation of F(ab)_2_ preparation containing pepsin with UNOsphere Q stationary phase under variuos pH conditions.

pH	Pepsin in unbound fraction	F(ab')_2_ yield [%]
4.0	+	97.6
5.0	-	89.2–93.0
6.0	-	86.2–91.3

Pepsin traces were detected by SDS-PAGE ("+" denotes presence and "-" denotes absence). Active principle yield [%] was calculated according to ELISA results.

#### Final polishing step

Optimal conditions for pepsin removal from F(ab')_2_ preparation determined in a batch mode, *i*.*e*. those that allow adsorption of pepsin traces and other residual acidic impurities exclusively, at the same time enabling passing of the active principle through anion-exchange resin without binding, were transferred to column chromatography in the flow-through mode. Concerning the loading of the sample, two different approaches were investigated. First, the digestion product (crude F(ab')_2_) was loaded to CIM QA disk either undiluted or 2-fold diluted with the binding buffer (20 mM MES + 0.15 M NaCl, pH 5.0). The SEC-estimated purity of F(ab')_2_ in the flow-through fraction was around 97%. Alternatively, the digestion product was first diafiltrated into binding buffer using a 50 kDa membrane (pure F(ab')_2_) (Figs 2E and 3A) and then submitted to final polishing which produced completely pure F(ab')_2_-based preparation (ultrapure F(ab')_2_ sample) without any loss (Figs 2F and 3A). The recovery for the CIM QA chromatography step was 100 ± 2.5% (*n* = 23) and the overall procedure yield was 77% (Table 1). The final product was completely aggregate-free (Fig 2F) and depleted from pepsin, as confirmed by SDS-PAGE and absence of a ''negative'' band at position corresponding to its molecular weight following silver staining (Fig 7B).

#### Efficacy of flow-through chromatographic final polishing in pepsin removal

The chromatographic behavior of pepsin was studied under conditions employed for the final F(ab')_2_ polishing by applying the enzyme preparation in quantities 10 times higher than that present in the digestion reaction mixture (Fig 4C). Thus, the potential of CIM QA disk for pepsin binding and removal was verified. Manufacturing by-products that affect pepsin purity did not bind (Fig 4D). No enzymatic activity could be detected in the flow-through fraction. The whole proportion of the enzymatically active material was retained on the column and it was subsequently eluted with 100% yield (Fig 4E). In comparison to the sample applied to column (8.67 ± 1.40 PU g^-1^), the enzymatic activity of the bound fraction was fully preserved (8.48 ± 0.72 PU g^-1^) when tested on haemoglobin.

#### MS and MS/MS analysis of the final product

**Fig 8:**
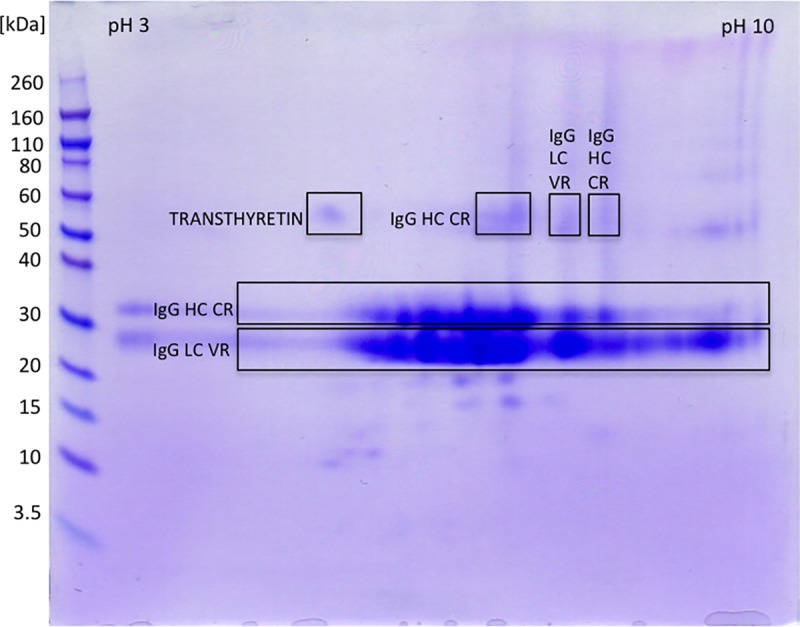
2D gel electrophoresis of the final product. In the first dimension F(ab')_2_ (350 μg) was focused using IPG strip under denaturing conditions (linear pH 3–10). Prior second dimension IPG strip was reduced, alkylated and loaded to a 4–12% gel. Proteins were detected with CBB R250 and identified by MS/MS analysis, as denoted (LC = light chain, VR = variable region; HC = heavy chain, CR = constant region). Molecular mass markers are at left side.

MS and MS/MS analysis of protein spots obtained by 2D gel electrophoresis confirmed a high purity of the final product. In 2D gel electrophoresis sample was intentionally overloaded so that even minor contaminants could be revealed. Predominantly segments of F(ab')_2_ fragments—different isoforms of heavy chain constant regions and light chain variable regions, were detected and identified (Fig 8, S1 Table). Of the rest discrete protein spots only transthyretin was confirmed while others remained unidentified.

#### Protective efficacies of IgG and F(ab')_2_ preparations

Neutralisation potencies of HHP and corresponding IgG/(Fab')_2_-based preparations from two possible manufacturing exit points, together with specific activities of their IgGs or F(ab')_2_ fragments, are summarised in Table 3. According to the lethal toxicity neutralisation assay in mice, each molecule of both active principle types exhibited comparable activity, proving that Fc portion is not relevant for neutralisation of venom toxins. Namely, 1 mg of IgGs from pure IgG sample is able to neutralise 1.81 LD_50_ venom doses. Because F(ab’)_2_ has 0.67 times lower molecular weight than IgG, 1 mg of F(ab’)_2_ contains 1.49 × higher number of active molecules. From that reason experimentally determined *Ƴ*(F(ab’)_2_) was multiplied by 1.49 prior specific activity of F(ab’)_2_ calculation. Purification factors of 2.2- and 3.6-fold, calculated as a ratio of specific activities of IgG or F(ab')_2_ product and HHP, respectively, were achieved.

**Table 3:** Neutralisation potencies of HHP and corresponding IgG/F(ab')_2_ determined *in vivo* together with specific activities. Purification factors obtained through manufacturing procedure are indicated.

	Plasma	Pure IgG	Ultrapure F(ab')_2_
^a^ *R* / LD_50_ mL^-1^	44.02 ± 15.94 (*n* = 7)	24.47 ± 2.37 (*n* = 3)	53.99 ± 3.65 (*n* = 2)
^b^ *γ*(IgG)/(F(ab')_2_) / mg mL^-1^	27.30 ± 2.28 (*n* = 9)	13.53 ± 1.13 (*n* = 12)	19.52 ± 0.2 (30.57 ± 0.31)* (*n* = 6)
^c^ Specific activity of active principle / LD_50_ mg^-1^	1.61 ± 0.6	1.81 ± 0.23	1.77 ± 0.12
^d^ *γ*(protein) / mg mL^-1^	58.19 ± 4.42 (*n* = 25)	14.78 ± 1.51 (*n* = 20)	19.52 ± 0.2 (*n* = 6)
^e^ Specific activity of sample / LD_50_ mg^-1^	0.76 ± 0.28	1.66 ± 0.23	2.77 ± 0.19
^f^ Purification factor	1	2.2	3.6

^a^ lethal toxicity neutralisation potency determined *in vivo*

^b^ IgG/F(ab’)_2_ mass concentration determined by ELISA

^c^ specific activity of active principle in the sample calculated as ratio of *R* to *γ*(IgG) or *γ*(F(ab')_2_), respectively

^d^ total protein mass concentration determined according to [32]

^e^ specific activity of the sample calculated as ratio of *R* to *γ*(protein)

^f^ purification factor calculated as ratio of specific activities of IgG or F(ab’)_2_ product and HHP

* Assumption of molecular weight reduction of the active principle due to Fc removal was taken into account. Results are given as mean +/- standard deviation.

## Discussion

**Fig 9:**
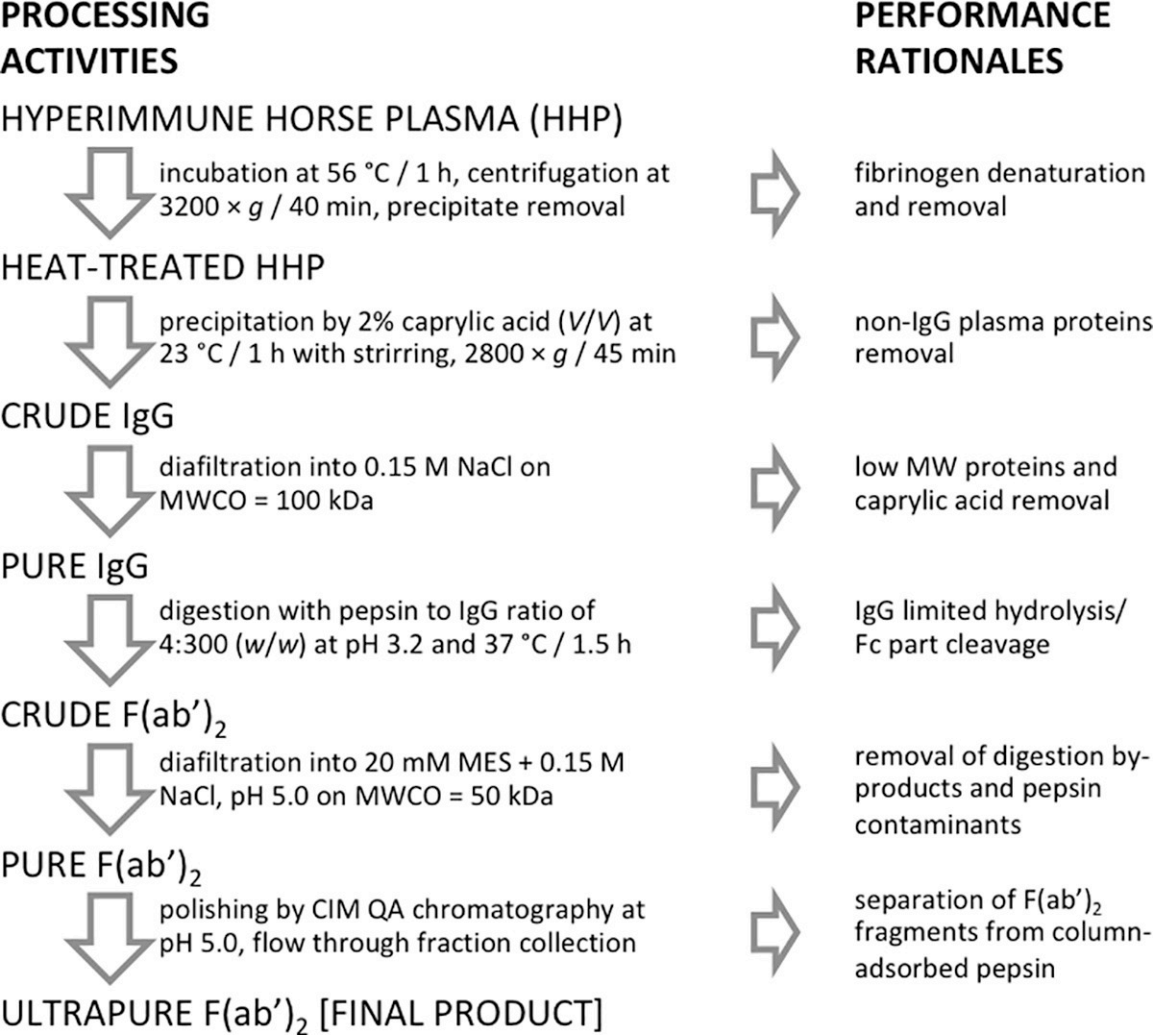
Flow sheet of downstream processing steps with corresponding samples and performance rationales.

The production of immunotherapeutics has always been a struggle of finding balance between retaining the potency of the product and reducing the appearance of its side effect-inducing properties. From the standpoint of antivenom manufacturing, consistent quality, safety and clinical efficacy are usually ensured through removal of the immunogenic Fc part of the IgGs previously fractionated from other plasma proteins and purification of the F(ab')_2_-based preparation from residual contaminants. Although deprived from innovative technological breakthroughs, our refining scheme (Fig 9) provides a finely tuned approach through which high yield and fulfillment of regulatory demands in the most straightforward way were achieved. Also, since the process efficiency has been supported with quantitative data, economic feasibility can be easily evaluated.

The development of the processing platform was demonstrated on HHP pool raised against *V*. *ammodytes* venom (S2 Fig). The emphasis was put on the active principle handling by preserving it in solution throughout the manufacturing procedure. Unwanted precipitation of IgGs was avoided by employing caprylic acid-mediated fractionation as a method introduced by Steinbuch and Audran [9]. This method is generally considered to represent a mild treatment. We found that the lowest volume ratio of precipitating agent with the most beneficial impact on purification fold was 2% (Fig 1). Preferential use of caprylic acid in the range of 1.5–3% has been described on a few other occasions, accompanied by the reports of boosting IgG purity to over 90% [18, 19, 36, 37]. Higher concentrations are usually associated with excessive tubidity and slower filtration rates [18, 36, 38, 39]. Fernandes *et al*. [38] noticed complete precipitation of albumin when 2% caprylic acid was employed. Our result was not in accordance with such finding since the same protein, although significantly depleted, proved as one of the most persistant contaminants in crude IgG sample, together with a low content of aggregates and < 13 kDa material, as detected by SEC.

Since caprylic acid precipitation or pepsin digestion do not change IgG subclass distribution, ELISA with a sample-specific correction of results [33] was found suitable for precise quantification of active principle. It has been chosen over other methods, such as SEC where overlapping of peaks or influence of protein type on surface area due to varying absorption and/or column adsorption characteristics is highly probable, leading to inaccurate result interpretation [40]. For instance, the resolution of SEC does not allow detection of potential impurities such as IgA, IgE and ceruloplasmin, which overlap with major peaks corresponding to IgG and albumin, as already emphasised [41]. Densitometric analysis of CBB or silver stained protein bands in SDS-PAGE, another method of purity profiling, also exhibits major drawback since intensity of developed color highly depends on amino acid composition and usually has a limited dynamic range [42]. According to ELISA-based calculations, yield of crude IgG preparation was almost 90% in regards to the input material (Table 1). The majority of loss occurred during the heat treatment step, probably due to entrapment of portion of IgG molecules into the denatured fibrinogen network. The literature in general lacks supportive data concerning recovery. For instance, Rojas *et al*. [7] reported 60% yield of caprylic acid-fractionated IgGs that has been considered as a satisfactory outcome [13].

Optimal conditions for pepsin digestion of equine IgGs, established on highly pure preparation from protein A chromatography, proved inadequate when crude IgG sample from precipitation step was employed as substrate because in the acidic environment residual caprylic acid provoked aggregation (Fig 6). Therefore, diafiltration as an intermediate step was introduced, also contributing to purity increase (Table 1), which was comparable to that reported by other groups [19, 37, 41]. ELISA-based yield assessment was additionally supported by *in vivo* assay, revealing purification factor of about 2-fold (Table 3). SEC analysis showed only 1–2% of aggregates. This is in line with literature as antivenoms derived from the whole IgGs purified by caprylic acid fractionation usually display low degree of aggregation [8, 12, 43]. In addition, protective efficacy of IgG preparation against *V*. *ammodytes* venom was congruent to that of the respective plasma pool (Table 3), indicating preservation of IgG subclasses and invariance of venom-specific antibody content through manufacturing process. As such, the pure IgG sample, although firstly regarded only as input material for subsequent antivenom manufacturing, later was recognised as possible production exit point as well due to compliance with regulatory frameworks concerning neutralisation potency and physicochemical profile [1].

Efficient pepsin cleavage aims for total IgG fraction breakdown and circumvention of over-digestion. The reaction should progress to the degree of Fc part removal only. In our protocol, complete removal yet limited hydrolysis was dependent on recognising 0.15 M NaCl as more appropriate for pepsin activity, adjusting enzyme to IgG ratio to 4:300 (*w*/*w*), setting pH to 3.2 and prolonging the incubation period to 1.5 h. We considered the avoidance of any kind of burden for the manufacturing process by introducing purity-compromising reagents such as pepsin in unrationaly high amounts. Namely, even shorter reaction time can be used but apparently seeks for higher enzyme concentrations to achieve comparable recovery levels [26]. Following digestion, apart from F(ab')_2_ fragments and by-products of enzymatic cleavage, a small amount of high molecular weight material near the exclusion limit (> 500,000 Da) was observed in SEC analysis (Fig 2D), but could not be seen by SDS-PAGE (Fig 3A, lane 6). This fraction might contain aggregated acidic low molecular weight fragments of Fc and/or other non-IgG fragments, as assumed by Jones and Landon [23] who noticed similar phenomenon following pepsin digestion of ovine serum. When diafiltration on 50 kDa membrane was employed for purity enhancement, basically only F(ab')_2_ product was detected (Figs 2E and 3A). Since no evidence of high molecular weight material was observed, dissociation of aggregates due to buffer exchange and washing out of released protein segments is very likely. Physicochemical profile of the pure F(ab')_2_ sample was equally good or better from that of some other final F(ab')_2_ products generated by various methodologies from hyperimmune plasma or serum on laboratory scale [18, 19, 26, 38, 41]. A recovery approaching 80% (Table 1) was favorably comparable to that reported for pepsin digested-hyperimmune antirabies serum prior its subsequent refinement by caprylic acid fractionation and diafiltration [38].

As already noticed by Jones and Landon [25], diafiltration was only partially efficient at removing pepsin (Fig 4A and 4B). Therefore, a third and final purification step was introduced. Polishing was performed by means of anion-exchange chromatography at pH 5.0 which allowed binding of residual acidic impurities, including the remaining pepsin content, and their segregation from F(ab')_2_ fragments which passed through the column and remained in solution. The methodology has already been successfully demonstrated on digestion product of ovine serum-derived IgG fraction [23, 25]. SEC profile indicated that completely pure and aggregate-free F(ab')_2_-based preparation was achieved (Figs 2F and 3A). In order to get a deeper insight on its purity or contaminant profile, as additional insurance of the final product quality, 2D gel electrophoresis and MS analysis were performed. Among traces of impurities only transthyretin was identified (Fig 8, S1 Table). Other low-abundance protein spots did not contain sufficient material for successful MS analysis and their identification failed. Exceptionally high purity of the final product is creditable to supplementing action of diafiltration and anion-exchange chromatography. Namely, diafiltration removes whole fraction of contaminating proteins/peptides from the pepsin preparation, which lack capability of binding to CIM QA disk, while anion-exchange chromatography depletes the other half of the enzyme material, latter remaining in retentate post-diafiltrationally. Overall yield of around 75% or higher (Table 1) was in the range of that reported for ovine serum as input material for fractionation with assumption that between 30 and 32 g of IgG per L are commonly found in immunised animal [19, 23]. ELISA-based calculation has been supported by the result of a lethal toxicity neutralisation assay in mice (Table 3). Functionality of the final product in terms of protective efficacy was comparable to that of the respective plasma pool and thus fully preserved. Thus, apart from quantity loss, reduction of neutralisation potency of F(ab')_2_ fragments due to denaturation induced by acidic conditions during pepsin digestion step can be excluded also, meaning that a good balance between pH level and reaction time was achieved. On the contrary, under quite similar pH/time conditions some authors noticed a decrease in F(ab')_2_ antigen-binding activity of around 35%, measured by competitive ELISA stated to be in close correlation with ED_50_ assay [44]. Others reported that performance of digestion at pH of 2.8–3.2 caused even 50% reduction of the plasma potency assessed in mice [45]. In addition, congruent protective efficacy of the final product and starting material points that no substantial IgG subclass loss and, consequently, redistribution of the venom-specific antibody content occurred.

In conclusion, fractionation of the venom-specific plasma was efficiently performed on laboratory scale by sequence of optimised purification steps—precipitation of unwanted proteins by caprylic acid, removal of precipitating agent from IgG-enriched fraction, pepsin digestion, diafiltration of the obtained F(ab')_2_ preparation and its final polishing by flow-through chromatography. During the whole process IgGs or F(ab')_2_ fragments were kept in solution, ensuring quality and, therefore, safety of the final product. Manufacturing protocol has been performed independently several times on two plasma pools of slightly different protective efficacy. Also, two analysts were involved. Refining scheme resulted in the completely pure, aggregate- and pepsin-free active principle with overall yield advantageously comparable to others so far reported. Suitability for larger scale production, as well as estimation of its cost-effectivenes, should be determined through additional study, together with stability, pre-clinical and clinical efficacy of the final product prepared according to optimised procedure.

## Supporting information

S1 Fig 2D gel electrophoresis of F(ab')_2_-based final product (same sample as in Fig 8) with annotations of protein spots subjected to MS/MS analysis. List of identified proteins is given in S1 Table.
(TIF)

S2 Fig Presentation of purification strategy with main research activities, goals and outcomes. (TIF)

S1 Table List of proteins detected in the final F(ab')_2_ sample. Proteins are denoted by numbers as in S1 Fig. Other protein spots were assigned based on PMF spectra overlapping or remained unidentified.
(DOCX)
