# Supplementary figures and images for: Refinement strategy for antivenom preparation of high yield and quality
Source: PLoS Negl Trop Dis. 2019 Jun 17;13(6):e0007431. doi: 10.1371/journal.pntd.0007431 (PMC6597126; doi:10.1371/journal.pntd.0007431)

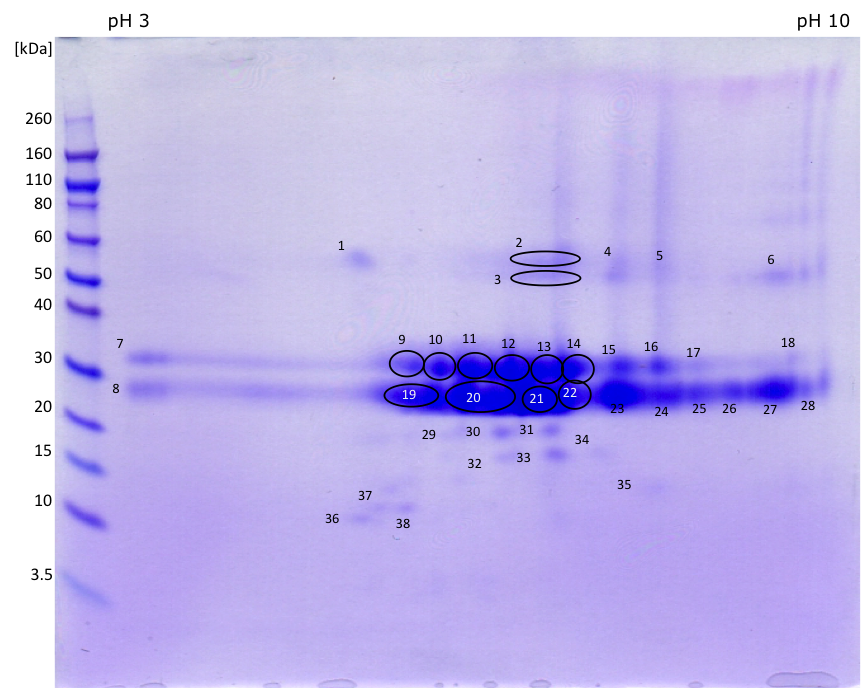

Supplement: S1 Fig — List of identified proteins is given in S1 Table. (TIF) [file pntd.0007431.s001.tif]

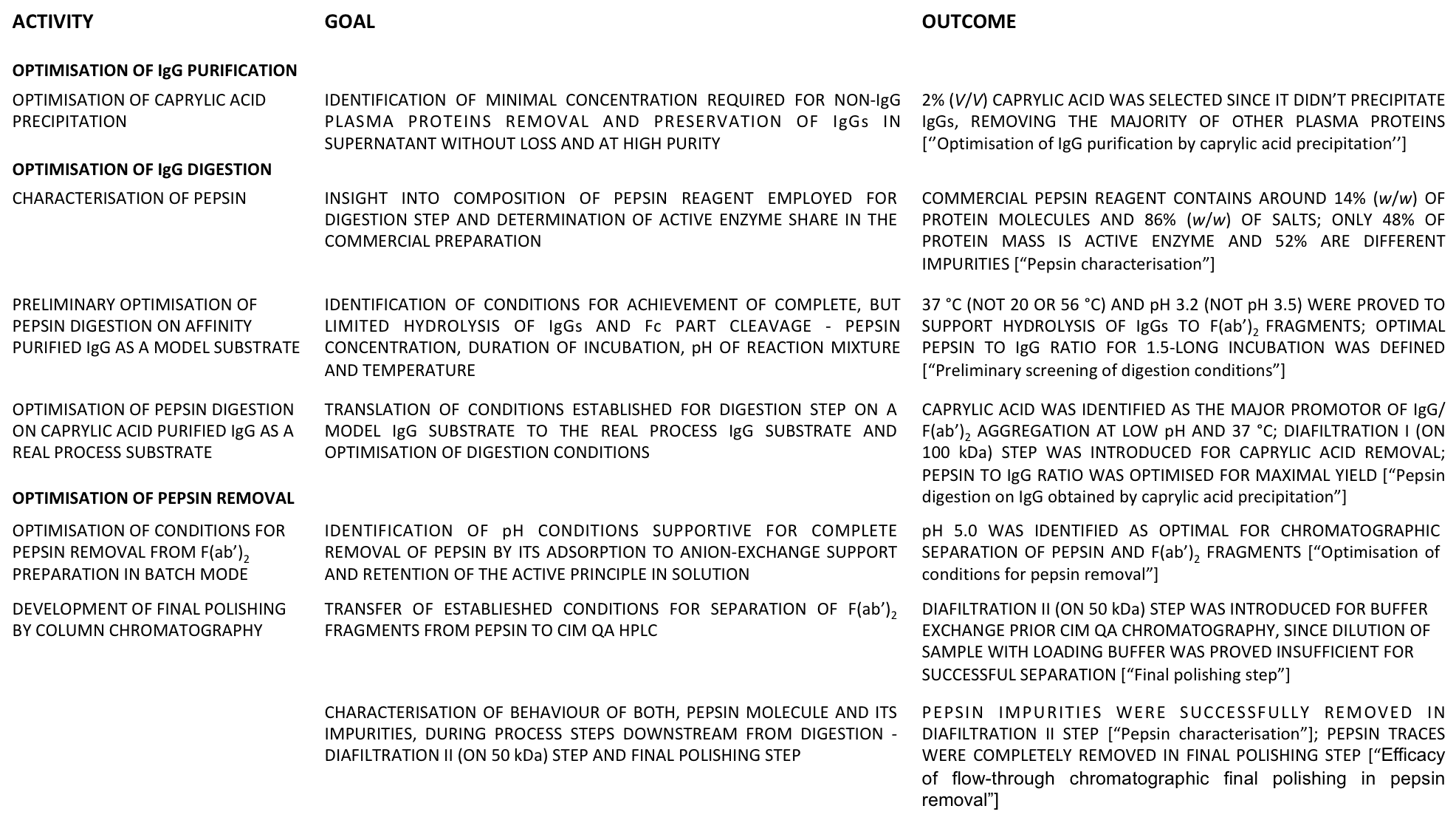

Supplement: S2 Fig — (TIF) [file pntd.0007431.s002.tif]

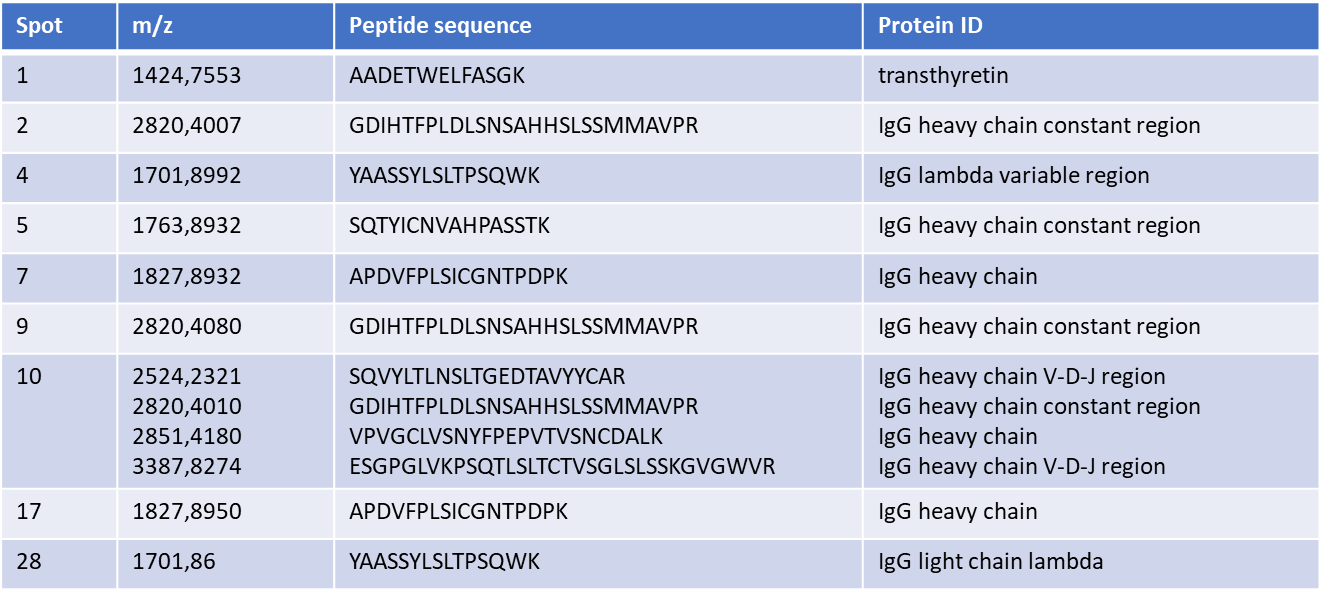

Supplement: S1 Table — Proteins are denoted by numbers as in S1 Fig. Other protein spots were assigned based on PMF spectra overlapping or remained unidentified. (DOCX) [file pntd.0007431.s003.docx]
